# Is There Any Need for Emergency Neuroimaging in Children With first Complex Febrile Seizure?

**Published:** 2020

**Authors:** Afagh HASSANZADEH RAD, Manijeh TABRIZI, Peyman DADASHZADEH, Vahid AMINZADEH

**Affiliations:** 1 Pediatric Diseases Research Center, Guilan University of Medical Sciences, Rasht, Iran.

**Keywords:** neuroimaging, convulsion, seizure

## Abstract

**Objectives:**

The current study aimed to assess the need for emergency neuroimaging in children with first CFC.

**Materials and methods:**

This is an analytic cross-sectional study conducted on children aged 6-60 months with first CFC. Data were gathered by a form that evaluates age, sex, imaging type, body temperature, and the duration of fever before convulsion, the duration and frequency of convulsion, and family history of FC. Data were analyzed via the Fisher Exact Test in SPSS version 19.

**Results:**

A total of 111 patients participated in this study with first CFC and mean age of 21.18±11.83 months. Regarding the type of CFC, the results showed that the highest and lowest frequencies belonged to multiple and multiple focal prolonged FC, respectively. Upper respiratory infection was the most common diagnosis. Also, 2 nonsignificant abnormal neuroimaging results were noted.

**Conclusion:**

Performing emergency neuroimaging in patients with first CFC was not mandatory in the absence of developmental disorders, abnormal neurologic examination, underlying neurological disorder, and head trauma. This is an important result in our country due to the lack of access to neuroimaging modalities in many hospitals, and the irradiation risk in childhood and its high cost.

## Introduction

Febrile convulsion (FC) is the most leading cause of neurologic disorders among infants and toddlers, accounting for 2-5% of children aged less than 5-year-old. It is also a chief cause of admission in the pediatric emergency ward.

FC may have circadian rhythm and seasonal variation. (1) It is more common among boys (2) and is defined as the occurrence of seizure in children aged 6-60 months with the temperature>38º C and negative history of afebrile seizure in the absence of central nervous system infection and/ or electrolyte imbalance (3).

It is broken down to simple and complex types. The simple type of FC is the common form of FC, 25-30% of which is diagnosed as complex febrile convulsion (CFC) (4) CFC is characterized as seizure which may be focal or multiple (>1 seizure) or lasting >15 minutes during febrile illness. It is accompanied with the increased risk of reoccurrence of FC, febrile status epilepticus, and epilepsy. (5) 

The American Academy of Pediatrics do not recommend performing routine neuroimaging for simple FC. (6) Regarding the documents which revealed no pathologic finding in children with SFC (7, 8), there is no consensus on performing emergency neuroimaging in patients with the first CFC and the previous investigations are limited. Furthermore, according to the lack of access to neuroimaging modalities in many hospitals of our country and due to the irradiation risk in childhood and high cost, we evaluated the need for emergency neuroimaging in children with the first CFC.

## Materials & Methods

This study is an analytic cross-sectional study conducted on children aged 6 -60 months with first CFC. It was approved by the Ethics Committee of Vice Chancellor of Research in Guilan University of Medical Sciences (NumberIR.GUMS.REC.1394.451, Date: 2 February 2015) The inclusion criteria encompassed all the patients with first CFC hospitalized in 17 Shahrivar Hospital, neurology ward in Rasht, Iran. The exclusion criteria were those who had the history of afebrile seizure, history of CFC, the history of underlying systemic diseases accompanied with convulsion or immune system condition, current neurosurgical intervention, and the existence of ventriculoperitoneal shunt, head trauma, abnormal neurological exam, and the decreased level of consciousness.

The positive radiologic findings encompassed important intracranial pathologic conditions which needed emergent neurosurgical or medical interventions and/ or any suspicious neuro-radiologic finding. The intracranial pathologic conditions were space-occupying lesion, intracranial hemorrhage, hydrocephaly, cerebral abscess, edema, etc. The emergent neurosurgical interventions included craniotomy, biopsy, or shunt insertion, etc. 

The two-month follow-up was indicated for patients who did not perform neuroimaging. The neuroimaging was reported by an expert neuroradiologist. 

Data were gathered by a form which assess age, sex, temperature, and the duration of fever before convulsion, the duration and the frequency of convulsion, family history of FC, and neuroimaging results. 

Data were reported by descriptive statistics (mean, standard deviation, frequency, and percent) and analyzed by Fisher Exact Test in SPSS version 19*.*

## Results

*111 patients with first CFC and mean age of* *21.18±11.83 months enrolled in this study. (Age* *range: 6 months- 5 years) .Most of the patients were boys (61.3%). (*Table 1*) The mean temperature during convulsion was* *38.29±0.61 centigrade.*

**Table 1:** Demographic characteristics of patients

*variables*	*NUMBER*	*PERCENT*
*SEX*		
*Boy* *girl*	*68* *43*	*61.3* *38.7*
*Age groups *		
*<1 year* *1-2 years* *2-3years* *>3 years*	*35* *54* *12* *10*	*31.5* *48.7* *10.8* *9*
*Consanguineous parents *		
*Yes* *no*	*23* *88*	*20.7* *79.3*
*Family history of FC*		
*yes* *no*	*94* *17*	*84.7* *15.3*
*Family history of epilepsy*		
*Yes* *no*	*11* *100*	*9.9* *90.1*

*Based on the subtypes of CFC in* Table 2 *, the results* *showed that the highest and the lowest frequency* *belonged to multiple frequency and multiple focal* *prolonged FC, respectively (40.5% & 1.8%,). In* *38 patients (34.23%), showed symptoms other* *than fever; the most common associated symptom* *was coughing (50%). Upper respiratory infection* *(89.2%) was the most frequent diagnosis (*Table 2*).* *Based on the importance of central nervous system* *infection in CFC, Lumbar puncture was performed* *in 41.4% of patients and negative result was noted.*

**Table 2:** Types, symptoms and etiology of fever in CFC

variables	number	percent
Types of CFC		
Multiple Focal Prolonged Multiple focal Multiple prolonged Focal prolonged Multiple focal prolonged	45 27 6 24 4 3 2	40.5 24.4 5.4 21.6 3.6 2.7 1.8
Associated symptom and sign		
Coughing Diarrhea Otalgia Sore throat Vomiting Failure to thrive	*19* *9* *3* *1* *5* *1*	*50* *23.7* *7.9* *2.7* *13.1* *2.6*
The etiology of fever		
Gastroenteritis Upper respiratory infection Acute otitis media Urinary tract infection Pneumonia	*7* *99* *3* *1* *1*	*6.3* *89.2* *2.7* *0.9* *0.9*

Among 111 patients with CFC, neuroimaging was performed in 72 patients (64.9%). Also, among these neuroimaging results, two cases of nonsignificant abnormality were observed (1.8%). (non-specific increased signal intensity in the occipital lobe and small arachnoid cyst). The results showed that there was no significant relationship between abnormal neuroimaging results and sex, age groups, type of CFC, symptoms, and signs, diagnosis, consanguineous parents, family history of febrile, and afebrile seizure. (Table 3).

**Table 3:** comparing characteristics based on normal and abnormal neuroimaging results

*Variables*	*Need CT*		*No- Need CT*		p-value*
	*Number*	*Percent*	*Number*	*Percent*	
*Sex*					
*Boy* *Girl* *Total*	*50* *22* *72*	*69.4* *30.6* *100*	*18* *21* *39*	*46.2* *61.3* *100*	*0.016*
*Age groups *					
*<1 year* *1-2 years* *2-3years* *>3 years* *Total*	*23* *35* *8* *6* *72*	*31.9* *48.6* *11.1* *8.3* *100*	*12* *19* *4* *4* *39*	*30.8* *48.7* *10.3* *10.3* *100*	*0.988*
Types of CFC					
Multiple Focal Prolonged Multiple focal Multiple prolonged Focal prolonged Multiple focal prolonged *Total*	*32* *16* *4* *16* *1* *2* *1* *72*	*44.1* *22* *5.6* *22.2* *1.4* *2.8* *1.4* *100*	*13* *11* *2* *8* *3* *1* *1* *39*	*33.3* *28.2* *5.1* *20.5* *7.7* *2.6* *2.6* *100*	*0.65*
Associated symptom and sign					
Coughing Diarrhea Otalgia Sore throat Vomiting Failure to thrive *Total*	*7* *5* *1* *0* *3* *3* *19*	*36.8* *26.3* *5.3* *0* *15.8* *15.8* *100*	*12* *4* *2* *1* *2* *0* *21*	*57.1* *19* *9.5* *4.8* *9.5* *0* *100*	*0.55*
The etiology of fever					
Gastroenteritis Upper respiratory infection Acute otitis media Urinary tract infection Pneumonia *Total*	*4* *66* *1* *1* *0* *72*	*5.6* *91.7* *1.4* *1.4* *0* *100*	*3* *33* *2* *0* *1* *39*	*7.7* *84.7* *5.1* *0* *2.6* *100*	*0.307*
*Consanguineous parents *					
*Yes* *No* *Total*	*12* *60* *72*	*16.7* *83.3* *100*	*11* *28* *39*	*28.2* *71.8* *100*	*0.152*
*Family history of FC*					
*yes* *no* *Total*	*12* *60* *72*	*16.7* *83.3* *100*	*5* *34* *39*	*12.8* *87.2* *100*	*0.591*
*Family history of epilepsy*					
*Yes* *No* *Total*	*9* *63* *72*	*12.5* *87.5* *100*	*2* *37* *39*	*5.1* *94.9* *100*	*0.21*

*Fisher Exact Test

## Discussion

FC is the most common type of seizure during childhood. Although the American Academy of Pediatrics did not recommend the emergency neuroimaging for patients with SFC, there is no general consensus for performing it in CFC.(9) 

In this study, most of the patients with CFC were 1-2 years old which was consistent with previous investigations. (9-11)

The family history of FC was positive in 84.7% of patients, which showed the effect of genetic factors on FC. This result was in accordance with the literature. However, inconsistent result was noted by Thakker et al. (10).

Multiple FC was mentioned as the most common type of CFC; multiple focal prolonged FC was the lowest frequent type of FC. This result was consistent with Kimia et al and Berzosa et al (9, 12).

The viral upper respiratory infection was the most common cause of CFC which was consistent with Kimia et al and Thakker et al. (9, 10)

In this study, 72 patients underwent neuroimaging. All had brain CT- scan and 30 of whom had both CT and MRI. The results mentioned two abnormal findings. As they did not have any neurologic sign and symptom except FC, no medical or surgical intervention during follow-up was performed. This was similar with previous investigations. (8-10, 12-13) 

Patients with no neuroimaging were followed up for two months and no symptom was noted. Similar result was noted by Teng et al. and Kimia et al. (8-9). 

Furthermore, there were no significant relations between the types of FC which was similar with previous investigation. (9) 

In this study, there was no significant relation between neuroimaging results with the associated signs and symptoms and the final diagnosis. 

However, in a study by Kimia et al., conducted for 50% of participants (526 patient), only four abnormal results were noted. These results were focal symptoms or altered level of consciousness. 

This study excluded those patients with altered level of consciousness and abnormal neurological sign or symptom except seizure. The different results were noted, regarding the lack of neurological sign. 

Furthermore, in a study by Berzosa et al. on 65 patients with CFC, only 1 patient had focal neurologic sign and the remaining patients had normal neuro imaging results (12). 

Limitations Short duration of assessment, little sample size, and parental stress during history taking were among the most notable limitations in this study. Although further multicenter studies are recommended, this prospective study enrolled more participants comparing previous investigations. 

In Conclusion, In this study, the results showed that performing emergency neuroimaging in patients with first CFC was not mandatory in the absence of developmental disorders, abnormal neurologic examination, underlying neurological disorder, and head trauma. The results of this study is important in our country because of the lack of access to neuroimaging modalities in many hospitals and the irradiation risk in childhood and high cost. 
